# Time to chemotherapy and oncofertility counseling in pediatric hematology/oncology patients: a single-center retrospective review

**DOI:** 10.3389/fonc.2026.1758483

**Published:** 2026-05-11

**Authors:** John Mariano, Hayley B. Flanagan, Kimberly H. Seymour, Nicole SanGiacomo, Nneka Ogbutor, Arman Niknafs, Brittany Hodges, Adam Evans, Andrea Baran, Jeffrey R. Andolina

**Affiliations:** University of Rochester Medical Center, Rochester, NY, United States

**Keywords:** adolescent and young adult (AYA), barriers, chemotherapy, fertility, oncofertility

## Abstract

**Background:**

The American Society of Reproductive Medicine and American Society of Clinical Oncology emphasize the importance of oncofertility counseling in pediatric cancer patients. Survivors often report inadequate attention to this crucial aspect of care. Balancing timely treatment initiation with oncofertility counseling presents challenges, including cost, age at diagnosis, information overload, and time constraints.

**Procedures:**

We performed chart abstraction of 265 pediatric patients diagnosed at the University of Rochester Medical Center (January 1, 2015–May 31, 2022). Hematologic malignancies made up 37.65% of the population. Median age was 7, with male predominance (56.2%). The majority of patients were Caucasian (80.7%).

**Results:**

Only 12.5% of the population had an oncofertility visit with reproductive endocrinology or urology and 42.4% had documented counseling with their oncology team. Sexual health was discussed in 2.6% of patients, and 12.1% of patients had documented Tanner staging. Hematologic malignancy patients had a lower time to systemic therapy (4.2 days) compared to extracranial solid tumors (14.8 days) and CNS tumors (97.8 days) when outliers were removed. Longer time to systemic therapy trended toward significance for oncofertility visits, and there was no statistical difference in oncofertility visits or primary team counseling rates based on disease severity.

**Conclusions:**

The time before chemotherapy initiation is a unique barrier to oncofertility counseling for pediatric patients with malignancy. While some patients will have retained fertility on current front-line protocols, patients with a high risk of relapse will receive therapy more likely to threaten fertility. The results of this study indicate that targeted intervention prior to relapse for this population may be a feasible approach.

## Introduction

Oncofertility counseling is a critical aspect for the care of pediatric cancer patients starting at diagnosis and spanning through survivorship. The clinical practice guidelines of the American Society of Clinical Oncology (ASCO) practicerecommend standardized fertility counseling for all adult and pediatric patients as soon as possible before receiving anticancer therapy to maximize options for fertility preservation (1). The American Society for Reproductive Medicine (ASRM) recommends programmatic requirements for comprehensive fertility preservation care, including rapid access, an interdisciplinary and collaborative medical team, and specific baseline laboratory testing (2). While these recommendations apply to both pediatric and adult patients, pediatric patients have a very specific set of challenges that make fertility preservation and sexual healthcare challenging.

Barriers to appropriate fertility preservation care in pediatric patients include high pressure to initiate therapy rapidly, potential financial expense for families, variable fertility preservation options in pre-pubescent vs. post-pubescent patients, need for counseling tailored to varying developmental stages, and the need for coordination around sedated baseline procedures (central line placement, echocardiogram, imaging, biopsies, lumbar punctures, etc.). These barriers have been described previously and have been the focus of a number of implementation studies in recent years (3).

Since the Research and Care Imperatives for Adolescents and Young Adults with Cancer Progress Review Group identified care gaps in adolescent and young adult patients with cancer in 2006, there has been abundant literature investigating fertility preservation in this population (4). Many targeted cross-sectional or retrospective analyses have investigated specific populations restricted by age, gender, or disease type, as summarized by Flink et al (5). While a number of these studies have estimated the prevalence of fertility counseling and/or successful preservation among patients receiving gonadotoxic therapies, many were limited by sample selection (i.e., gender, age, disease type, etc.) (6–14). Other studies have sought to assess the perspective of cancer survivors, who often report limited or ineffective pre-treatment fertility counseling despite their expressed desire for more dedicated support in this area (15–21). Despite potential for recall bias and loss to follow-up impacting these findings, these qualitative data indicate an area of supportive oncologic care in need of improvement. Notably, while patients recognize the importance of fertility preservation discussions, as evidenced by a group of adolescent female cancer survivors who indicated interest in treatments to help preserve fertility when feasible, these patients also expressed a lack of willingness to postpone cancer therapy (22).

Most oncologists also appreciate the need for fertility preservation counseling and management. However, many report feeling underequipped to do so due to lack of education and/or formalized resources (23–26). Numerous single-institution reports have highlighted the benefits of standardized oncofertility programs. These studies have measured improvement in documentation of fertility discussions, referrals placed for fertility services, and ultimate pursuit of fertility preservation in pediatric or young adult patients (27–33).

The University of Rochester Medical Center Pediatric Hematology/Oncology program sees between 60 and 80 new oncologic patients annually. It historically has not had a formalized multidisciplinary oncofertility program established. The longstanding structure has been oncology–provider-specific referral to reproductive endocrinology and infertility or urology for persons with ovaries or testes, respectively, based on perceived risk of regimen to fertility or upon prompting from patients or families. The standardized chemotherapeutic consent form includes a clause referencing a risk for fertility generally with the receipt of chemotherapy, but the risk of infertility or effects to sexual health has not been rigorously outlined historically.

At the time of this study, the University of Rochester Medical Center did not maintain a formal, multidisciplinary oncofertility program. Oncofertility care was instead coordinated by individual oncology providers based on their assessment of each patient’s risk for fertility impairment or when prompted by patient or family inquiry. Referrals for fertility preservation evaluation were sent directly to reproductive endocrinology and infertility specialists.

Fertility preservation options presented to patients were primarily determined by age, pubertal status, and clinical urgency. Post-pubertal males were generally offered sperm banking when sufficient time allowed prior to therapy initiation. For post-pubertal females, possible options included oocyte or embryo cryopreservation, though these require additional time for ovarian stimulation and coordination with reproductive endocrinology. Prepubertal patients had access only to experimental or investigational options, such as ovarian or testicular tissue cryopreservation, which were not routinely available at our center until 2019 when the American Society of Reproductive Medicine guidelines recommended ovarian tissue cryopreservation in pre-pubertal patients or those unable to receive oocyte/embryo cryopreservation.

Standard chemotherapy consent forms included general language referencing potential impacts of chemotherapy on fertility; however, a detailed risk assessment for infertility or broader discussions on sexual health were not standardized during the study period. All referrals and counseling were left to the discretion of the primary oncology provider.

In contrast, recent proposed standardization of pediatric oncofertility programs incorporate multidisciplinary care with a clear standardized operating procedure for rapid-access pathways to fertility preservation procedures, defined expectations for developmentally appropriate counseling, timing of assessments and interventions within the cancer journey, and resources for exploration of insurance coverage and financial support among other factors (34).

This study illustrates a single-center experience characterizing real-world oncofertility counseling and referrals in a pediatric hematology/oncology practice without an established oncofertility program in the era prior to utilization of standardized risk assessment tools. Unlike prior work, it formally quantifies the relationship between time to systemic therapy and oncofertility services across all tumor types in a real-world setting that is reflective of historical baselines, contributing pragmatic data to inform the extent and magnitude of influence of oncofertility guideline-based programmatic implementation on outcomes.

## Materials and methods

We conducted a retrospective review of pediatric hematology/oncology patients at the University of Rochester Medical Center from January 1, 2015 to May 31, 2022 using manual chart abstraction.

Electronic medical record data were collected with the following variables: demographic information (age, gender, race, ethnicity, zip code), diagnosis, date of pathologic diagnosis, date of systemic chemotherapy initiation, and disease risk factors (stage, risk stratification, etc.). Oncofertility documentation by the treating oncologist was defined as any explicit note in the oncology provider’s documentation indicating that potential treatment-related impacts on fertility were discussed, oncofertility documentation from the fertility provider which was a binary outcome suggestive of a successful completed outpatient encounter, and Tanner staging. We did not formally grade the depth of counseling as the level of listed details varied too greatly across providers.

### Outcomes

The primary outcomes were (1) completion of an oncofertility documentation by the treating oncologist as outlined above and (2) documentation of completion of an oncofertility visit with a reproductive endocrinology/infertility specialist or urologist. We did not systematically capture whether the patients underwent specific fertility preservation procedures as completion of the procedure cannot be definitively confirmed by the electronic medical record. While it was possible to infer this, we aimed to maintain data integrity and omit this assessment.

### Statistical analysis

Patient and disease characteristics were summarized using medians for continuous variables and counts and proportions for categorical variables. Time to systemic therapy was calculated in days from the date of diagnosis to the date of systemic chemotherapy initiation and summarized using median within diagnosis group. For this analysis, treatment initiation refers exclusively to the start of systemic chemotherapy and does not include surgery or radiation due to limitations in available verification of the latter variables. Fertility counseling outcomes were summarized using counts and proportions, overall and within diagnosis group. Multivariate logistic regression was used to assess the association of time to systemic therapy, age, patient’s sex, diagnosis, and disease risk with the fertility counseling outcomes. All *p*-values are two-sided, and all hypothesis tests were conducted at the 0.05 level of significance. SAS v9.4 (SAS Institute, Inc., Cary, NC, USA) was used for all analyses. It is worth noting that standard fertility preservation procedures were not routinely available for pre-pubertal patients at our center during the study period; however, because pubertal status was not available for all patients and pubertal status can vary with age, we have accepted this as a limitation in the analysis.

### Disease risk determination

Disease risk determination was defined by utilizing a standardized approach to electronic medical record for the abstraction team. The chart was searched for the term risk and separated by primary oncologist risk assignment, and for lymphoma or solid tumor patients, stage was chart searched and collected when documented. We considered the patients to have high-risk disease characteristics if they were stage 3 or 4 or assigned as with high-risk disease by the primary oncologist.

## Results

Hematologic malignancies comprised 37.65% of the cohort, with acute lymphoblastic leukemia (15.43%) and acute myeloid leukemia (7.72%) being the most prevalent. The median age was 7, with a male predominance (56.2%). The patients were predominantly Caucasian (80.6%) (**Tables 1**, **2**).

**Table 1:** Patient demographics.

Age:	
Median Age (Yrs):	7.42
Age 10 or Older (%)	42.21
Age 9 or Less (%)	57.79
Sex (%):	
Female	43.94
Male	56.06
Ethnicity (%):	
Hispanic	4.94
Non-Hispanic	80.99
Other	1.52
Unknown	12.55
Race (%):	
Asian or Pacific Islander	2.28
Black or African American	9.89
Other	4.94
Unknown	2.28
White or Caucasian	80.61

^N^ *total number = 265*.

**Table 2:** Patient diagnosis distribution.

Diagnosis	Percent of total (Count)
B-Cell ALL	18.9% (50)
T-Cell ALL	3% (8)
B-Cell LLy	1.1% (3)
T-Cell LLy	1.1% (3)
AML	7.6% (20)
Burkitt lymphoma	2.6% (7)
DLBCL	1.89% (5)
Hodgkin lymphoma	5.3% (14)
Lymphoma, other	1.1% (3)
NLPHL	3% (8)
Diffuse midline glioma	3.8% (10)
Low grade glioma	5.3% (14)
Medulloblastoma	2.3% (6)
Ewing sarcoma	4.5% (12)
Germ cell tumor	3.8% (10)
Hepatoblastoma	1.1% (3)
Neuroblastoma	4.1% (11)
Osteosarcoma	3% (8)
Other	12.8% (34)
Rhabdomyosarcoma	7.6% (20)
Sarcoma, other	1.1% (3)
Wilm’s Tumor (Nephroblastoma)	4.9% (13)

^N^ total number = 265.

Hematologic malignancy patients and extracranial solid tumors generally had a shorter time between pathologic diagnosis and treatment initiation (14.4 and 14.8 days, respectively) compared to CNS tumors (97.8 days). When outliers such as nodular lymphocyte predominant Hodgkin’s lymphoma (NLPHL) and B-cell lymphoblastic lymphoma were removed from the hematologic malignancy group, this interval was shortened even further to 4.2 days (**Table 3**). Additionally, diagnoses that generally require an immediate inpatient admission for treatment initiation following diagnosis such as acute leukemia and Burkitt lymphoma had an expectedly very short time (1–4 days) to systemic therapy, while those diagnosed and treated primarily in the outpatient setting such as sarcomas or Hodgkin’s lymphoma had a less acute initiation of therapy (7–20 days). Given that a number of CNS tumors may have had periods of post-operative monitoring or observation prior to systemic therapy initiation, these patients’ protracted period prior to therapy initiation correlated as expected to the disease course.

**Table 3:** Time to therapy by diagnosis.

Diagnosis	Time in days (IQR)
B-Cell ALL	1 (0-2)
T-Cell ALL	1 (0.5-6.5)
B-Cell LLy	16 (13-167)
T-Cell LLy	3 (0-35)
AML	1.5 (0-2.5)
Burkitt lymphoma	4 (2-13)
DLBCL	7 (3-8)
Hodgkin lymphoma	13 (8-23)
Lymphoma, other	3 (1-3)
NLPHL	24.5 (19.5-38)
Diffuse midline glioma	32 (3-133)
Low grade glioma	36 (31-45)
Medulloblastoma	33.5 (29-40)
Ewing sarcoma	14.5 (7-22.5)
Germ cell tumor	14.5 (7-38)
Hepatoblastoma	7 (7-36)
Neuroblastoma	10 (5-19)
Osteosarcoma	8 (7-11)
Other	24 (6-60)
Rhabdomyosarcoma	20 (8.5-29.5)
Sarcoma, other	11 (6-25)
Wilm’s Tumor (Nephroblastoma)	14 (8-21)

^N^ total number = 265.

With respect to oncofertility outcomes, only 12.5% of the patient population saw reproductive endocrinology or urology for an oncofertility visit, and 42.4% had documented oncofertility discussions with their oncology team, not including consent forms which were not universally readily available. Overall proportions are shown with confidence intervals in **Supplementary Figure 1**. Sexual health was discussed in only 2.6% of patients, and only 12.1% of the patients had documented Tanner staging before treatment initiation. **Supplementary Figures 2**, **S3** display the distribution of time from diagnosis to systemic chemotherapy initiation, stratified by specialist visit and primary-team counseling status, respectively, across diagnostic categories. The rates of both specialist visits and primary team counseling did increase in the populations with a median age at diagnosis that typically occurs in the adolescent and young adult range, as defined by the World Health Organization, of 15 to 39 years old—for example, oncofertility visits and primary team oncofertility discussions were relatively high in Hodgkin’s lymphoma (42.9% and 53.8%), diffuse large B-cell lymphoma (40.0% and 60.0%), and germ cell tumors (70% and 50%) (**Table 4**), all of which are more commonly diagnosed in adolescent and young adult populations.

**Table 4:** Rate of oncofertility visits and oncofertility discussions with primary oncologists.

	Oncofertility urology/REI visit		Oncofertility discussion with primary oncologist	
Diagnosis:	No	Yes	No	Yes
B-Cell ALL	48	1	20	30
T-Cell ALL	7	1	4	4
B-Cell LLy	3	0	1	2
T-Cell LLy	3	0	2	1
AML	18	2	10	10
Burkitt lymphoma	6	1	3	4
DLBCL	3	2	2	3
Hodgkin lymphoma	8	6	6	7
Lymphoma, other	2	1	2	1
NLPHL	8	0	4	4
Diffuse midline glioma	10	0	10	0
Low grade glioma	12	2	12	2
Medulloblastoma	6	0	6	0
Ewing sarcoma	7	1	6	6
Germ cell tumor	3	7	5	5
Hepatoblastoma	3	0	3	0
Neuroblastoma	10	1	6	5
Osteosarcoma	7	1	4	4
Other	32	2	26	8
Rhabdomyosarcoma	17	3	11	9
Sarcoma, other	3	0	1	2
Wilm’s Tumor (Nephroblastoma)	12	1	8	5

Adjusted for age, gender, and disease risk, time to systemic therapy trended toward significance for oncofertility outcomes both in terms of oncofertility specialist visits and primary team oncofertility counseling. Interestingly, these rates trended inversely in that a longer time to systemic therapy led to increased odds of an oncofertility visit, while a shorter time to systemic therapy led to decreased odds of primary team oncofertility counseling. There was insufficient evidence of an association of disease risk and patient sex with either outcome. The only factor that was significantly associated with counseling outcome was older age at diagnosis, with an adjusted OR of 1.28 (*p* < 0.0001, 95% CI: 1.17–1.40) and 1.06 (*p* = 0.009, 95% CI: 1.01 – 1.10) for oncofertility subspecialist visits and primary team oncofertility counseling, respectively (**Table 5**).

**Table 5:** Odds of oncofertility visit or primary team oncofertility counseling.

	Odds ratio (OR)	P-value (95% CI)
Oncofertility visit:		
Time to therapy	1.11	0.052 (0.99, 1.23)
Age at diagnosis	1.28	<0.0001 (1.17, 1.40)
Female (vs Male)	0.45	0.09 (0.18, 1.12)
High risk (High Risk Clinical Features/Cytogenetics or Stage III/IV vs others)	0.57	0.22 (0.24, 1.39)
Primary Team Oncofertility Counseling:		
Time to therapy	0.85	0.09 (0.70, 1.03)
Age at diagnosis	1.06	0.009 (1.01, 1.10)
Female (vs Male)	1.31	0.30 (0.79, 2.17)
High risk (High Risk Clinical Features/Cytogenetics or Stage III/IV vs others)	1.09	0.75 (0.65, 1.82)

## Discussion

Barriers to oncofertility care have been extensively described since the identification of this supportive care need in the oncologic population. While the narrow time window prior to therapy initiation is often cited, the specific effects of time to chemotherapy initiation have not been studied in depth. In this study, we provide a retrospective assessment of time to chemotherapy initiation and oncofertility counseling in a medium-sized pediatric hematology/oncology center without a formalized oncofertility program prior to the institution of modern risk-stratification tools.

We demonstrate that providers instinctively tend to counsel and refer post-pubescent patients despite guidelines indicating that this should be universally applied across the age spectrum. Additionally, even with low rates of primary oncologist oncofertility counseling, when this counseling occurred, there were even lower rates of specialist referrals. In part, this is likely due to the more limited and often more invasive options for fertility preservation that are available for pre-pubescent patients (35). These highlight the need for a standardized approach to oncofertility counseling and documentation by the primary oncology team to meet the goal of ensuring that all patients receive adequate and appropriate information prior to chemotherapy initiation.

A major limitation of this study is our inability to classify patients by detailed therapy-based infertility risk. Although we know that specific exposures such as hematopoietic cell transplant, pelvic or gonadal radiation, and high-dose alkylating regimens carry substantial gonadal risk, regimen-level data (including cumulative doses and radiation fields) were not consistently extractable from the retrospective record for the entire cohort without extensive re-abstraction and risk of misclassification. As a result, we used disease risk/severity as a pragmatic but imperfect proxy.

In the absence of reliable information regarding infertility, we used disease risk severity as a potential marker not only for upfront therapy intensity but for the potential for disease recurrence which often leads to much more intensive therapy in the pediatric oncology landscape. Disease risk is related to, but not synonymous with, infertility risk: patients with “high-risk” disease may receive more intensive, gonadotoxic therapy but also face greater urgency to initiate treatment, whereas some “standard-risk” regimens can still meaningfully affect fertility. Consequently, our findings should be interpreted primarily as describing system-level barriers—time to systemic therapy and absence of standardized oncofertility infrastructure—rather than therapy-risk-adjusted appropriateness of individual referral decisions. We did not see that disease severity had any impact on the rates of oncofertility counseling or subspecialist referral, implying that despite the anticipated therapy intensity, referral rates did not increase.

As a limited sensitivity check, during this study period, patients with Ewing sarcoma, Burkitt lymphoma, and Hodgkin’s lymphoma would almost uniformly receive moderately to high gonadotoxic therapy at our institution. In this small subset, oncofertility counseling and reproductive specialist visit rates remained low (50% and 16.7%, 57.1% and 14.3%, and 53.8% and 42.9%, respectively), suggesting that the system−level barriers that we described extend even to patients with clearly elevated infertility risk.

Importantly, these data were inclusive of the survivorship period, so this care gap occurred not only prior to initial therapy but potentially within the window of remission. This is of particular importance for patients with hematologic malignancies in which frontline therapy itself may not result in infertility or sexual health disruption, whereas second-line therapy, including allogenic stem cell transplant, would be very likely to do so. This highlights another layer of need in oncofertility standardization that extends from upfront counseling to continued monitoring and revisited assessment during the survivorship period, which is highlighted by the current Children’s Oncology Group Long-Term Follow-Up Guidelines (36).

Other important limitations of the study include the retrospective design, lack of longitudinal fertility preservation outcomes, lack of discrete measures of sexual development at time of counseling, and challenges with uniform utilization of the international classification of diagnosis codes (ICD) in the electronic medical record. Additionally, documentation itself was determined to be binary as either present or not; however, in this quantitative analysis, important qualitative factors such as provider comfortability, duration of counseling, and factor that triggered counseling cannot be assessed. As documentation and standardization of oncofertility care within the electronic medical record improves, future studies can better delineate the relationship between time to systemic therapy and onocofertility outcomes within the context of current risk-stratification models and more widely available fertility preservation techniques.

These findings, however, underscore the need for standardized, guideline-concordant documentation of fertility counseling and referrals. We propose, at a minimum, that documentation should (1) provide appropriate developmental/sexual history, (2) explicitly state whether and to what magnitude fertility impairment is likely to occur, (3) point out the specific preservation options discussed, (4) record whether a referral to a reproductive specialist was offered and/or completed, (5) and capture the patient and/or family preferences in decision-making.

Embedding this into structured electronic medical record templates and care coordination tools with concordant resource support for a multidisciplinary team is vital—for example, the CRISP tool developed to simplify the workflow for cyclophosphamide equivalent dosing can be incorporated into electronic medical record (EMR) to flag oncofertility referrals at specific risk levels after receipt of chemotherapy in periods of remission or survivorship (37). Additionally, incorporation of best practice advisories within the EMR, which appear to significantly improve referral rates, is another feasible intervention that can be readily pursued (38). Importantly, coupling a multidisciplinary team with these measures of automated referral has been shown to improve both types of documentation, fertility preservation outcomes and patient-reported outcomes (39).

In conclusion, this is the first study to characterize the relationship of time to systemic therapy and oncofertility outcomes across tumor types in a real-world pediatric hematology/oncology population prior to initiation of standardized oncofertility risk-assessment tools. It can serve as a foundation for prospective interventional studies aimed at assessing the magnitude of outcome change resulting from instituting a standardized operating procedure both for upfront oncofertility counseling and referral as well as longitudinal oncofertility counseling during survivorship. Additionally, it highlights a key area of need in the pre-pubescent population with an achievable goal of improving counseling despite the challenges of fertility preservation in this population.

## Data Availability

The raw data supporting the conclusions of this article will be made available by the authors, without undue reservation.
