# Supplementary material for: Time to chemotherapy and oncofertility counseling in pediatric hematology/oncology patients: a single-center retrospective review
Source: Front Oncol. 2026 May 11;16:1758483. doi: 10.3389/fonc.2026.1758483 (PMC13199080; doi:10.3389/fonc.2026.1758483)
Supplement: Supplementary file 1 [file DataSheet1.docx]

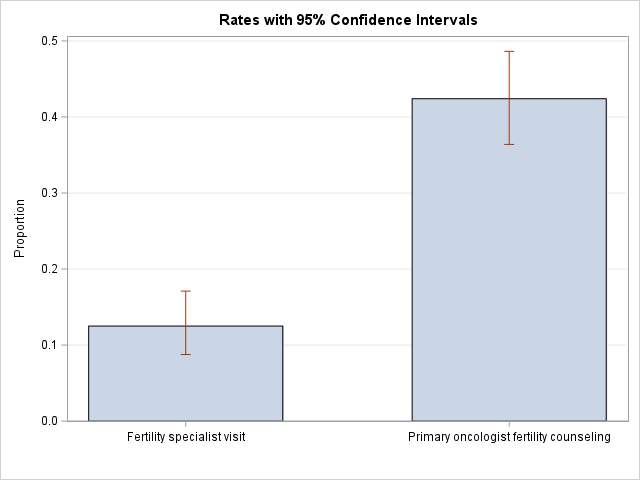


1. **Supplemental Figure S1:**
   Overall proportion of fertility specialist visits and primary oncology team fertility counseling with 95% confidence intervals


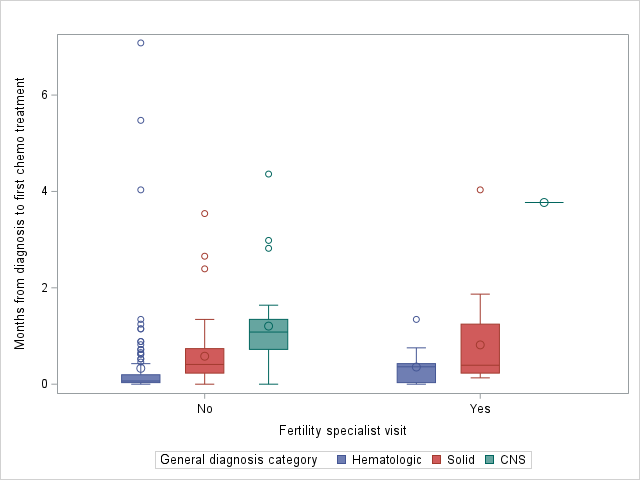


1. **Supplemental Figure S2**
   Time from pathologic diagnosis to systemic therapy initiation in months separated by fertility specialist visit status and diagnostic group. Boxplots show median, interquartile range, and 95% confidence intervals.


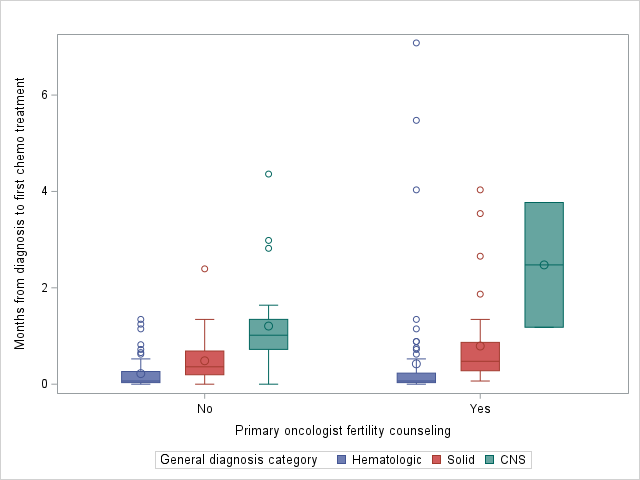


1. **Supplemental Figure S3**
   Time from pathologic diagnosis to systemic therapy initiation in months separated by primary oncologist fertility counseling and diagnostic group. Boxplots show median, interquartile range, and 95% confidence intervals.
